# A Clinical Case

**Published:** 1890-04

**Authors:** Wm. Steinrauf

**Affiliations:** St. Charles, Mo.


### A CLINICAL CASE.

Wm. Steinrauf, M. D., St. Charles, Mo.

About ten months ago I was called to see E. B., a child of eight years, who had been sick for many weeks with chronic pleurisy. The pleura was filled with pus. Two physicians of the old school had attended the case during this time, and had dosed the child liberally with cathartics, Croton Oil to the chest, Quinine to keep down the fever, and Opium to procure sleep. They introduced an aspirating needle into the pleural cavity, but the pus was so thick it would not run. Death stared the child in the face, as he was reduced to a mere skeleton. The family now decided to send for a homœopathist, as he could do no harm if he did no good, and the boy would die in any event.

I was called to see the child on a fine April morning. The pulse was 130, and the temperature 103°. There was extreme dullness on the whole right side of the chest, a terrible cough, with a muco-purulent, horribly offensive expectoration; very exhausting night-sweats; no appetite; in short, the child was *in extremis*.

Now, here was a perplexing case. What could we do for such a child? There were no particular indications for any remedy, yet something must be done. I therefore dropped ten pellets of Sulphur$^{30x}$ into a tumbler half-full of water, and ordered a sip to be given to the patient every four hours, and the parents to report in three days. At the end of this period the fever had disappeared, the bowels had moved, the cough became less, and sleep returned. No more medicine was given. When I saw the boy again in four weeks, there was almost complete reabsorption of the purulent secretion in the thorax.

The patient has since continued well.

